# Comparative transcriptome analysis identifies candidate genes related to seed coat color in rapeseed

**DOI:** 10.3389/fpls.2023.1154208

**Published:** 2023-03-09

**Authors:** Mingwei Guan, Xiangtian Shi, Si Chen, Yuanyuan Wan, Yunshan Tang, Tian Zhao, Lei Gao, Fujun Sun, Nengwen Yin, Huiyan Zhao, Kun Lu, Jiana Li, Cunmin Qu

**Affiliations:** ^1^ Integrative Science Center of Germplasm Creation in Western China (CHONGQING) Science City and Southwest University, College of Agronomy and Biotechnology and Academy of Agricultural Sciences, Southwest University, Chongqing, China; ^2^ Academy of Agricultural Sciences, Southwest University, Chongqing, China; ^3^ Affiliation Engineering Research Center of South Upland Agriculture, Ministry of Education, Chongqing, China

**Keywords:** *Brassica napus* L., yellow trait, transcriptome, flavonoid pathway, protein-protein interaction

## Abstract

Yellow seed coat in rapeseed (*Brassica napus*) is a desirable trait that can be targeted to improve the quality of this oilseed crop. To better understand the inheritance mechanism of the yellow-seeded trait, we performed transcriptome profiling of developing seeds in yellow- and black-seeded rapeseed with different backgrounds. The differentially expressed genes (DEGs) during seed development showed significant characteristics, these genes were mainly enriched for the Gene Ontology (GO) terms carbohydrate metabolic process, lipid metabolic process, photosynthesis, and embryo development. Moreover, 1206 and 276 DEGs, which represent candidates to be involved in seed coat color, were identified between yellow- and black-seeded rapeseed during the middle and late stages of seed development, respectively. Based on gene annotation, GO enrichment analysis, and protein–protein interaction network analysis, the downregulated DEGs were primarily enriched for the phenylpropanoid and flavonoid biosynthesis pathways. Notably, 25 transcription factors (TFs) involved in regulating flavonoid biosynthesis pathway, including known (e.g., KNAT7, NAC2, TTG2 and STK) and predicted TFs (e.g., C2H2-like, bZIP44, SHP1, and GBF6), were identified using integrated gene regulatory network (iGRN) and weight gene co-expression networks analysis (WGCNA). These candidate TF genes had differential expression profiles between yellow- and black-seeded rapeseed, suggesting they might function in seed color formation by regulating genes in the flavonoid biosynthesis pathway. Thus, our results provide in-depth insights that facilitate the exploration of candidate gene function in seed development. In addition, our data lay the foundation for revealing the roles of genes involved in the yellow-seeded trait in rapeseed.

## Introduction

Rapeseed (*Brassica napus* L., AACC, 2n = 38) is an important oilseed crop worldwide, serving as a source of edible vegetable oil and feed meal (Saeidnia and Gohari, 2012; Carruthers et al., 2017; Kaur et al., 2020). Increasing seed oil content is an important breeding objective in rapeseed. Improving the seed oil content of rapeseed by 1% is equivalent to a 2.3–2.5% increase in seed yield (Wang, 2004). The vegetable oil from rapeseed is excellent for human health (Lu et al., 2011; Liu et al., 2016). Therefore, the demand for high-quality rapeseed has risen sharply. Yellow-seeded rapeseed has many favorable qualities over black-seeded varieties, such as higher oil and protein contents, lower fiber content, and fewer pigments and polyphenols (Tang et al., 2010; Chao et al., 2022). Therefore, the yellow-seeded trait is a major breeding objective for rapeseed.

Seeds are specific sites of oil accumulation in *B. napus*. Seed development in rapeseed, like the model plant Arabidopsis (*Arabidopsis thaliana*), can divided into two phases: embryogenesis and seed maturation (Ruuska et al., 2002; Locascio et al., 2014). Carbohydrates, proteins, triacylglycerol (TAG), and pigments are important reserves in developing seeds. Many genes involved in the biosynthetic pathways of these compounds have been identified in Arabidopsis (White et al., 2001; Zhang M. et al., 2009; Lu et al., 2020; Seok et al., 2021). Among these pathways, the flavonoid biosynthesis pathway is a ‘model’ for secondary metabolite production. Flavonoids perform different functions in plants, including color formation and resistance to abiotic and biotic stresses (Nishihara and Nakatsuka, 2011; Chezem and Clay, 2015; Davies et al., 2018). Much effort has focused on revealing the flavonoid biosynthesis pathway involved in seed color formation using *Arabidopsis TRANSPARENT TESTA* (*TT*) mutants, such as mutants of *TT1*-*TT19*, *TTG1*, and *TTG2* (Albert et al., 1997; Dong et al., 2001; Bharti and Khurana, 2003; Kerhoas et al., 2006; Fu et al., 2007; Stracke et al., 2007; Yoshida et al., 2008; Cutanda-Perez et al., 2009; Baudry et al., 2010; Appelhagen et al., 2011; Chen et al., 2013; Pang et al., 2013; Stein et al., 2013; Zhang et al., 2013; Gesell et al., 2014; Ichino et al., 2015; Gonzalez et al., 2016; Zhang et al., 2017). Numerous genes involved in the formation of seed coat color have also been identified in *Brassica* species. For example, *TRANSPARENT TESTA GLABRA 1* (*TTG1*) shares the same functions in *B. rapa* as its Arabidopsis counterpart, controlling both hairiness and seed coat color traits in this crop (Zhang J.F. et al., 2009). The bHLH transcription factor (TF) gene *TT8* regulates the accumulation of proanthocyanidins (PAs) and controls seed coat color in *Brassica* species (Li et al., 2012); silencing of *TT1* genes and knockout of *TT2* homologs altered seed coat color in *B. napus* (Lian et al., 2017; Xie et al., 2020). In *B. napus*, flavonoid biosynthesis pathway genes, including the P-type H^+^-ATPase genes (*AHA10*), *TT10*, *TT12*, and *TTG1*, are located within the quantitative trait locus (QTL) region for seed coat color on chromosome A09 (Liu et al., 2005; Badani et al., 2006; Fu et al., 2007; Xiao et al., 2007; Yan et al., 2009; Zhang et al., 2011; Stein et al., 2013; Zhang et al., 2013; Qu et al., 2015). In addition, several candidate genes (e.g., MYB2, MYB3, MYB4, TT8, and MYBL2.1) involved in PA accumulation have been identified in *Brassica* species using transcriptomic analysis, providing important information for identifying candidate genes for seed coat color (Hong et al., 2017; Jiang et al., 2019; He et al., 2022). However, the molecular mechanisms controlling the yellow-seeded trait in rapeseed remain largely unexplored.

To explore the dynamic regulation of seed coat color formation, we comprehensively investigated the dynamic changes in gene expression during seed development in yellow- and black-seeded rapeseed by performing multistage comparative transcriptomic analysis. Many candidate genes were uncovered, such as *KNOTTED-like homeobox of Arabidopsis thaliana 7* (*KNAT7*), *SEPALLATA 2* (*SEP2*), *G-box binding factor 6* (*GBF6*), *SEEDSTICK* (*STK*), and *TTG2*, which play important roles in regulating seed development and seed coat color formation in rapeseed. Notably, some candidate genes, including *BnaA02g17180D*, *BnaC06g22430D*, *BnaA07g21710D*, *BnaA05g00070D*, and *BnaA04g01810D*, were differentially expressed in black-seeded vs. yellow-seeded *B. napus* during seed development. These results increase our understanding of the gene networks involved in yellow trait formation during seed development in rapeseed, which will facilitate breeding efforts for this trait.

## Materials and methods

### Plant materials

Seven inbred rapeseed (*Brassica napus*) lines, four yellow-seeded lines (GH06, 16G15, 16G16, and 16G47), and three black-seeded lines (ZY821, 16G48, and 17G56) were grown in experimental field in Beibei (106.38°E, 29.84°N), Chongqing, China. Inbred lines GH06 (Y1) and ZY821 (B1) are typical yellow- and black-seeded *B. napus* lines (Qu et al., 2013), respectively. Two groups of near-isogenic lines developed by Chongqing Rapeseed Technology Research Center (CRTRC) were planted during 2019–2020 in the field in CRTRC in Beibei, Chongqing, China: 16G15 (Y2), 16G16 (Y3) and 16G48 (B2); and 17G56 (B3), and 16G47 (Y4). At the flower initiation stage, individual flowers were marked with different colored wool to ensure that seed development could be dated exactly. Subsequently, seeds were sampled at 15, 30, and 50 DAP (days after pollination). At each stage, samples from five individual plants were pooled as a biological replicate, immediately frozen in liquid nitrogen, and stored at −80°C until total RNA extraction.

### RNA extraction, cDNA library construction, and sequencing

For each sample, 3 μg RNA was used as input material for cDNA library preparation. Total RNA was extracted from the samples using an RNAprep Pure Plant Kit (Tiangen Biotech, Beijing, China) following the manufacturer’s instructions, and index codes were added to attribute sequences to each sample. First-strand cDNA was synthesized using random hexamer primers. Second-strand cDNA synthesis was then performed using DNA Polymerase I and RNase H. The remaining overhangs were converted into blunt ends using a MicroPoly (A) Purist Kit (Ambion, USA). The library fragments were purified with an AMPure XP system (Beckman Coulter, Beverly, MA, USA). Size-selected, adaptor-ligated cDNA was digested with 3 μl USER Enzyme (NEB, USA) at 37°C for 15 min and then at 95°C for 5 min. The fragments were amplified by PCR using Phusion High-Fidelity DNA Polymerase, Universal PCR Primers, and Index (X) Primer. Following quality checking, the RNA was used for library construction using a NEB Next Ultra RNA Library Prep Kit for Illumina with an insert size of 300 bp. The Illumina HiSeq X was used, which generates short reads of 150 bp in PE mode.

### Identification of differential gene expression (DEGs)

After removing low-quality reads, Illumina sequencing reads were mapped to the *B. napus* Darmor-bzh reference genome (http://www.genoscope.cns.fr/brassicanapus) (Chalhoub et al., 2014) using HISAT with default settings (Kim et al., 2015). The bam files of uniquely mapped reads were used as inputs for HTseq, and FPKM (fragments per kilobase of transcript per million mapped reads) values were calculated to measure the expression levels of genes (Roberts et al., 2011; Anders et al., 2015). The Pearson correlation coefficient between biological replicates was calculated based on the normalized expression levels of log2(FPKM + 1). DEGs were recognized by Cuffdiff with a cut-off of log2 fold change (FC) ≥ 1 and a false discovery rate (FDR) ≤ 0.05 (Wang et al., 2009; Love et al., 2014).

### Enrichment analysis of DEGs

Clustering analysis was performed using the TCseq clustering package, and PCA was performed using the Pheatmap package. Transformed and normalized gene expression values with log2 (FPKM + 1) were used for hierarchical clustering.

GOseq was used to obtain significant GO terms for the DEGs in the significant modules (Young et al., 2010), and the clusterProfiler package in R was used to perform KEGG (Kyoto Encyclopedia of Genes and Genomes) enrichment analysis of DEGs in the modules: *P* < 0.02 was selected as the cut-off value.

### Identification of hub genes and sub-networks associated with seed color

Hub genes, which are highly interconnected with nodes in a module, are functionally significant. The top 25% of genes evaluated by Cytoscape_v3.7.2 with the CytoNCA (BC, Betweenness centrality; CC, Closeness centrality; DC, Degree centrality) application of the STRING database (http://www.string-db.org) were considered to be hub genes. Hub genes were identified among DEGs between black- and yellow-seeded rapeseed. Protein–protein interaction (PPI) networks were established with STRING, with a cut-off confidence score > 0.4 (medium confidence). Cytoscape_v3.7.2 was then used to calculate the sub-modules of the DEG interaction network. Hub genes were identified according to BC, CC, and DC (Shannon et al., 2003; Chin et al., 2014). The top 25% of genes were ultimately identified as crucial genes based on the centrality values of genes in the PPI network.

### Weight gene co-expression networks analysis (WGCNA) and integrative gene regulatory network (iGRN) analysis to identify target genes

The RNA-seq data were analyzed to construct gene co-expression networks using the R package WGCNA (Langfelder and Horvath, 2008). To reduce noise, genes with criterion FPKM ≤ 3 in each sample were excluded. In total, 21 samples were used for analysis, including 7 lines each at the early, middle, and late stages of seed development. The following parameters were used to identify each gene module: weighted network, unsigned; hierarchical clustering tree, dynamic hybrid tree cut algorithm; power, 30; and minimum module size, 250.

Gene regulation is a dynamic process in which TFs play an important role in controlling spatiotemporal gene expression (De Clercq et al., 2021). To identify TFs closely related to seed color, iGRN was used to predict candidate genes. iGRN is used to determine the enrichment of TFs associated with (i.e., regulating) a set of input genes. Enrichment statistics were computed using hypergeometric distribution combined with Benjamini–Hochberg correction for multiple hypotheses testing (with a q-value cut-off of 1e-3). Hub genes confirmed by PPI and Cytoscape_v3.7.2 were used to predict candidate TFs. The results of analysis, combined with the classification of hub genes by WGCNA, were jointly used to predict TFs that are closely associated with the formation of seed color in rapeseed. The expression values of the TFs were calculated using FPKM of materials with the same seed color. A heatmap was generated using the R package Pheatmap (scale = “row”, cluster_row = T) (Diao et al., 2018).

## Results

### Overview of sequencing data analysis

Through library construction and sequencing, 158.95 million reads in yellow- and black-seeded *B. napus* during seed development were generated using the Illumina sequencing platform, and deposited in the NCBI database (Accession No., PRJNA931458). An average of 89.26% of the reads were mapped to the *B. napus* Darmor-*bzh* reference genome (version 4.1, http://www.genoscope.cns.fr/brassicanapus/data/; Chalhoub et al., 2014) using HTseq version 2.2, and the expression profiles of the genes were quantified in terms of FPKM. Of these mapped reads, an average of 83.32% were uniquely mapped and used to calculate normalized gene expression levels. Detailed information about the transcriptome sequencing data is given in **Table 1**. Based on the *B. napus* reference genome (http://www.genoscope.cns.fr/brassicanapus), 89,136 expressed genes were detected during seed development. After eliminating 31,089 genes with lower expression levels (FPKM < 1) in all samples, the 58,047 remaining genes were used for further analysis.

**Table 1:** Summary statistics of RNA-Seq results during seed development in *B. napus*.

Sample	Total reads	Total mapped reads	Uniquely mapped reads	Mapped paired-end reads
Y1-15DAP	117,534,018	90.23%	84.51%	81.27%
Y1-30DAP	130,975,684	90.84%	84.67%	83.05%
Y1-50DAP	135,283,922	91.15%	80.63%	82.78%
B1-15DAP	116,634,942	89.24%	83.21%	80.03%
B1-30DAP	132,568,802	90.56%	84.38%	82.85%
B1-50DAP	127,441,128	91.28%	80.88%	82.52%
Y2-15DAP	64,070,908	89.75%	85.41%	82.96%
Y2-30DAP	49,766,158	87.05%	82.77%	78.96%
Y2-50DAP	46,453,248	87.32%	79.97%	77.04%
Y3-15DAP	53,759,758	89.41%	84.79%	83.29%
Y3-30DAP	54,646,278	84.22%	78.81%	75.99%
Y3-50DAP	49,294,400	88.66%	81.73%	79.32%
B2-15DAP	48,872,142	88.60%	84.02%	81.77%
B2-30DAP	47,936,028	86.30%	80.82%	77.79%
B2-50DAP	56,744,446	86.91%	80.03%	76.88%
Y4-15DAP	55,991,424	91.34%	86.76%	84.54%
Y4-30DAP	67,242,064	91.30%	86.91%	84.31%
Y4-50DAP	47,889,758	91.39%	85.76%	84.61%
B3-15DAP	58,565,488	89.82%	85.26%	82.66%
B3-30DAP	61,333,478	90.17%	85.63%	83.13%
B3-50DAP	66,537,336	89.01%	82.69%	82.01%

Total reads indicates all clean reads in every sample; total mapped reads indicates the ratio of mapped reads in every sample; uniquely mapped reads indicates the ratio of reads in each sample that mapped to a single location; mapped paired-end reads indicates the ratio of paired-end reads that mapped to the genome. DAP, days after pollination.

### Identification and annotation of differentially expressed genes during seed development

**Figure 1:**
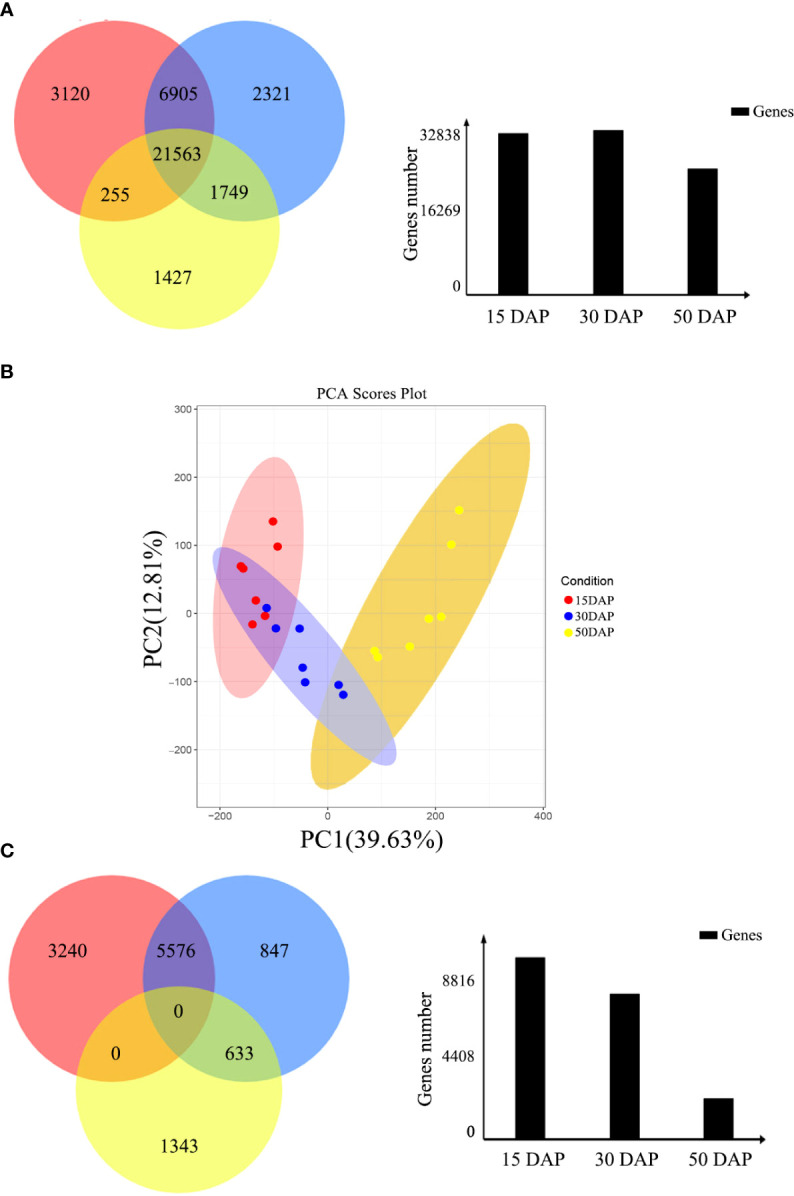
Identification of differentially expressed genes during seed development. **(A)** Venn diagram of the number of genes expressed during seed development. The red, blue, and yellow circles represent the early, middle, and late stages of seed development, respectively. The numbers represent the number of genes. The bar chart shows the total number of genes expressed during the early (15 DAP), middle (30 DAP), and late (50 DAP) stages of seed development. **(B)** Cluster dendrogram showing three distinct developmental stages: the early, middle, and late stages of seed development. **(C)** Venn diagram of DEGs expressed during seed development. The red, blue, and yellow circles represent the early, middle, and late stages of seed development, respectively. The numbers represent the number of DEGs. The bar chart shows the total number of DEGs expressed during the early (15 DAP), middle (30 DAP), and late (50 DAP) stages of seed development.

In this study, 31,843, 32,538, and 24,944 genes with high expression levels (FPKM ≥ 1) were identified at 15, 30, and 50 days after pollination (DAP), respectively (**Figure 1A**). Of these genes, 21,563 were expressed throughout seed development, and 3,120, 2,321, and 1,427 were specifically expressed at 15, 30, and 50 DAP, respectively. Based on the clustering analysis and principal component analysis (PCA) of the differentially expressed genes (DEGs), these DEGs were divided into three groups, which are consistent with the stages of seed development: early (E, 15 DAP), middle (M, 30 DAP), and late (L, 50 DAP) stages (**Figure 1B**).

**Figure 2:**
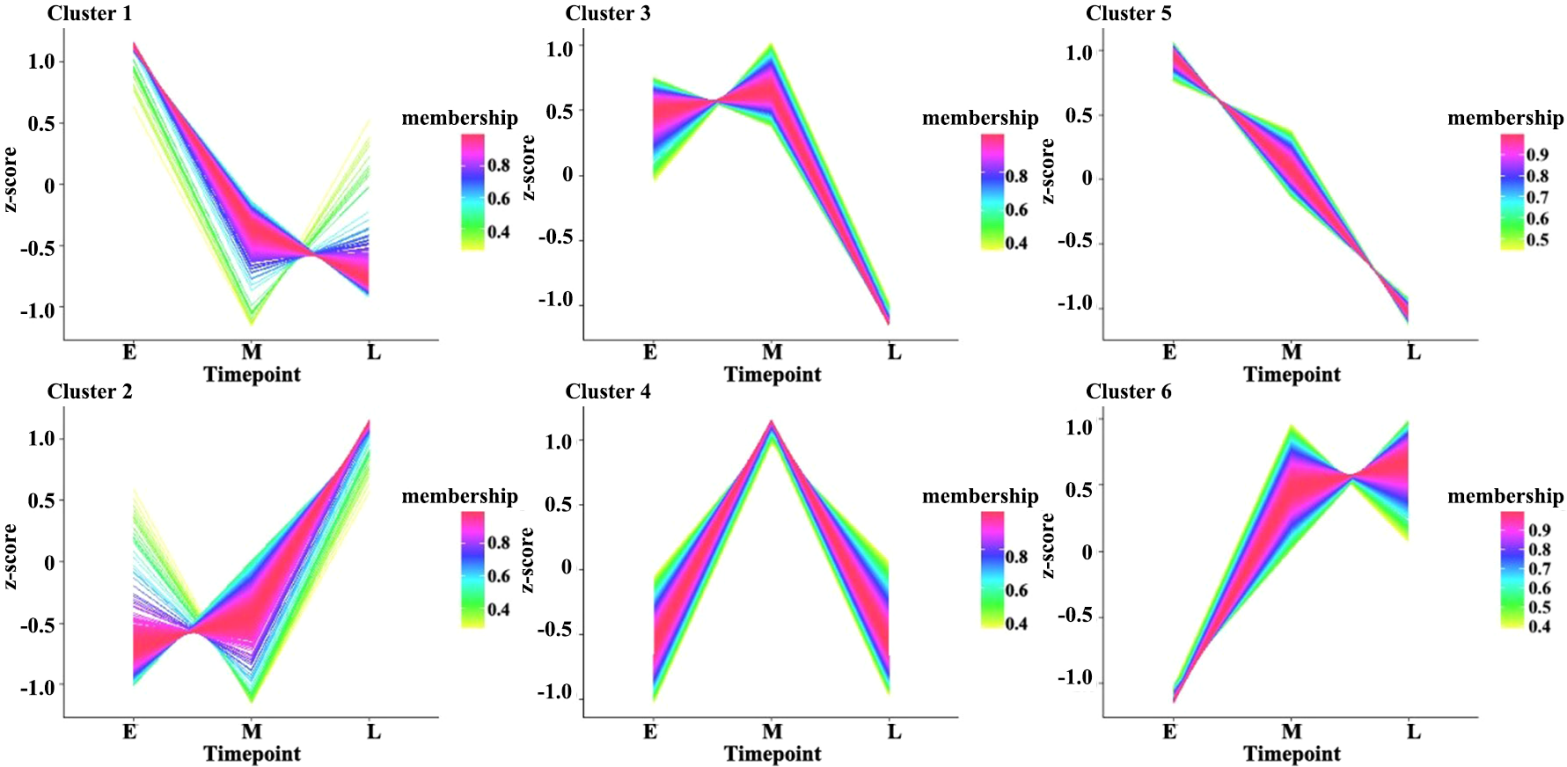
Clustered gene expression profiles in developing seeds. The clusters were defined based on the temporal expression profiles of genes using K-means clustering in R. The Y-axis represents the standardized FPKM values of genes, and E, M, and L on the X-axis represent seed samples at 15, 30, and 50 DAP, respectively.

Moreover, the expression levels of the DEGs during the three stages of seed development were investigated *via* pairwise comparisons. Using DEGseq and DESeq, the DEGs with adjusted log2 fold change (FC) > 1 and q-value ≤ 0.05 were further investigated. The results of DEG analysis between materials are shown in **Table S1**. The results showed that 3,240, 847, and 1,343 DEGs were specifically expressed during the early, middle, and late stages of seed development, respectively, in all materials (**Figure 1C**). However, other genes did not show stable differences in expression among materials during seed development. In addition, a total 1,224 DEGs (FPKM ≥ 1), including 438 genes with high expression levels (FPKM ≥ 10) during seed development, had stable expression levels throughout development. These genes could be core genes for seed development in rapeseed. These DEGs were grouped into six clusters by K-means clustering (TCseq package; **Figure 2**). The expression of DEGs in clusters 1 and 5 peaked during early seed development, while the expression of DEGs in clusters 2 and 6 peaked during late seed development, and genes in the other clusters were expressed at their highest levels during the middle stage of seed development (**Figure 2**).

**Figure 3:**
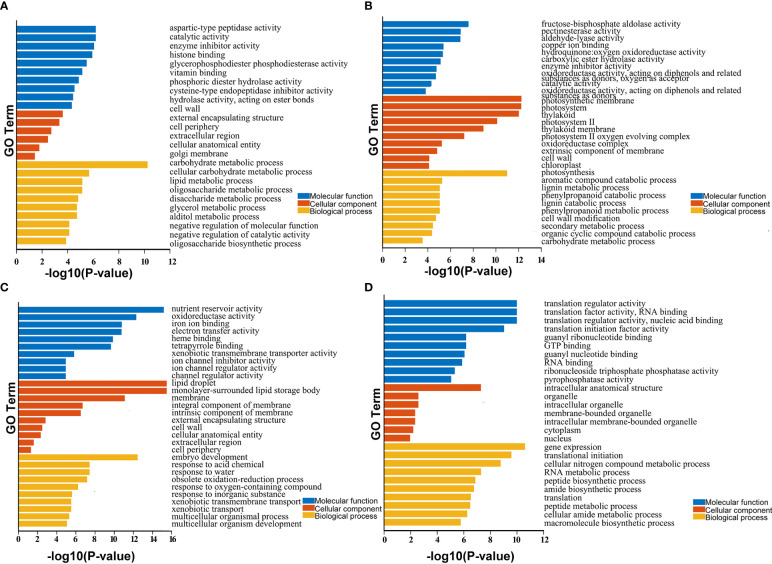
GO enrichment analysis of identified DEGs and core genes in developing seeds. **(A–D)** show enriched GO terms in seeds at 15, 30, and 50 DAP and 438 core genes with stable, high expression levels during seed development. Only significant categories (top 10 sorted by P-value in MF, CC, and BP) are displayed.

Besides, the functions of these DEGs were predicted using Gene Ontology (GO) and KEGG enrichment analysis. The GO terms were divided into the biological process (BP), cellular component (CC), and molecular function (MF) categories (**Figure 3**). As shown in **Figure 3**, the DEGs were enriched in different GO terms during the three stages of development. For example, the most highly enriched terms during early seed development included carbohydrate metabolic process in the BP category and catalytic activity and enzyme inhibitor activity in the MF category. During the middle stage of seed development, the most highly enriched GO terms included photosynthesis in the BP category and photosynthetic membrane and photosystem in the CC category. During late seed development, the most highly enriched GO terms included embryo development in the BP category, lipid droplet and monolayer-surrounded lipid storage body in the CC category, and nutrient reservoir activity in the MF category (**Table S2**). KEGG enrichment result showed that the pathways involved mainly carbohydrate, starch and sucrose metabolism in the early seed development; photosynthesis proteins and photosynthesis metabolism in the middle stage; and cutin, suberine and wax biosynthesis, transporters, cytochrome P450 and lipid in the late stage, etc. (**Table S3**). These results suggest that these DEGs play different roles during seed development in *B. napus*.

GO enrichment analysis of the 438 core genes with stable, high expression levels during seed development revealed that these genes were clustered into 89 GO terms. The top three terms were translation regulator activity, translation factor activity, and RNA binding and translation regulator activity in the MF category; intracellular anatomical structure, organelle, and intracellular organelle in the CC category; and gene expression, translational initiation, and cellular nitrogen compound metabolic process in the BP category (**Table S2**). KEGG enrichment result showed the most highly enriched in the pathway of translation factors, protein families: genetic information processing and spliceosome, etc. (**Table S3**). These results suggest that these DEGs may play crucial roles in translation and the regulation of seed development in *B. napus*.

### Identification of DEGs between the yellow- and black-seeded rapeseed

**Figure 4:**
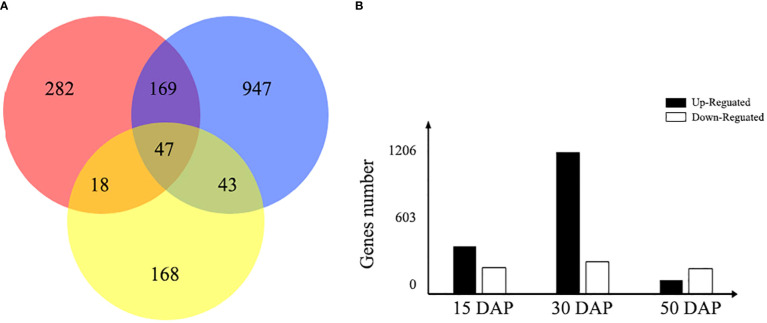
Venn diagram of the number of DEGs expressed during seed development. **(A)** The red, blue, and yellow circles represent the early, middle, and late stages of seed development, respectively. The number represents the number of DEGs between yellow- and black-seeded rapeseed. **(B)** The bar chart shows the total number of DEGs expressed during early (15 DAP), middle (30 DAP), and late (50 DAP) seed development. Black bars indicate upregulated DEGs in yellow seeds, and white bars indicate downregulated DEGs in yellow seeds.

To identify changes in gene expression related to the yellow-seeded trait, the expression patterns of DEGs were further investigated between yellow- and black-seeded lines during seed development (15, 30, and 50 DAP). In total, 516, 1,206, and 276 DEGs between yellow- and black-seeded rapeseed were obtained in the early, middle, and late stages of seed development, respectively (**Figure 4**). The highest number of DEGs was observed in the middle stage of seed development (1,206 DEGs, including 982 upregulated and 224 downregulated DEGs in yellow-seeded materials), while fewer DEGs were detected in the late stage of seed development (276 DEGs, including 96 upregulated and 180 downregulated DEGs in yellow-seeded materials). The DEGs in the middle stage of seed development may play important roles in seed color formation. In addition, we identified 47 common DEGs during all three stages of seed development, including 27 upregulated and 20 downregulated genes (**Table S4**).

### Identification of hub genes associated with seed color *via* PPI network analysis

**Figure 5:**
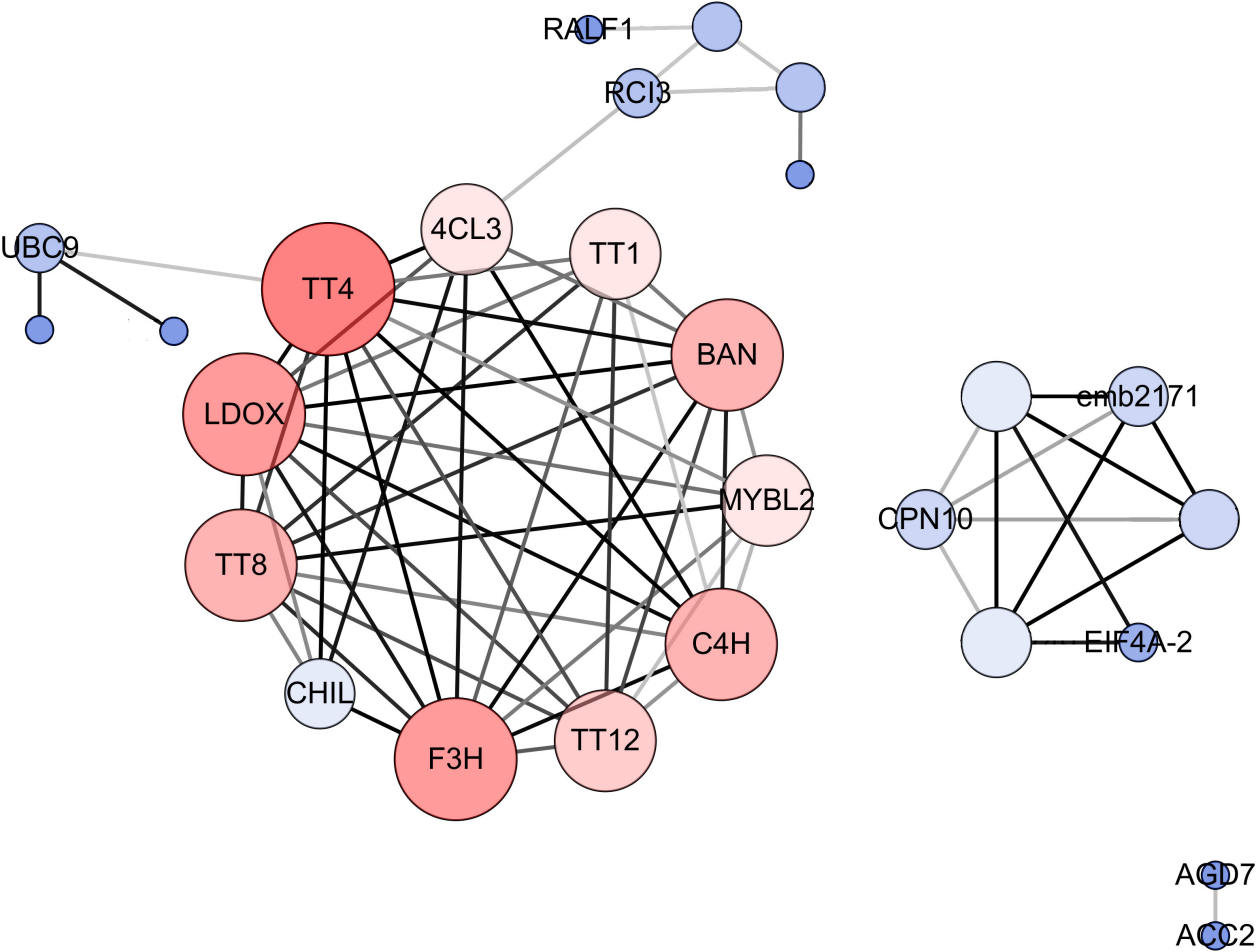
PPI networks for hub genes involved in *B. napus* seed color. The size and color depth of the circle represent the degree of center, and the color depth of the line represents the degree of correlation. Crucial genes were identified based on eigenvector centrality (EGC), degree centrality (DC), and closeness centrality (CC), which were calculated using the Cytoscape_v3.7.2 plug-in CytoNCA.

To explore the molecular mechanism of seed color formation in *B. napus*, protein–protein interaction (PPI) networks were constructed using the STRING online database, with parameters including a minimum required interaction score > 0.4 (medium confidence). The upregulated and downregulated DEGs in yellow-seeded *B. napus* seeds at three stages of development were submitted to the STRING database for predicting PPIs and construct PPI networks (**Figure S1**; **Table S5**). Strikingly, the PPI networks of downregulated DEGs were highly consistent, especially during the middle and late stages of seed development; these genes are mainly associated with the proanthocyanidin and flavonoid biosynthesis pathways (**Table S5**). To further elucidate the interactions of these genes, these downregulated and repeatedly detected DEGs in the middle and late stages of seed development were submitted to the STRING database and reconstructed the PPI networks. We then selected the top 25% of genes as hub genes following evaluation by Cytoscape_v3.7.2 with the CytoNCA (BC, CC, DC) application (**Figure 5**). These hub genes, including *TT* (*TT1*, *TT4*, *TT8*, *TT12*, and *TT16*), *BANYULS* (*BAN*), *Cinnamate-4-hydroxylase* (*C4H*), *AHA10*, *Leucoanthocyanidin dioxygenase* (*LDOX*), *MYB-LIKE 2* (*MYBL2*), and *Phenylalanine ammonia-lyase* (*PAL*), were downregulated in yellow- vs. black-seeded rapeseed (**Table S5**), suggesting they might play important roles in seed color formation in rapeseed.

### Weight gene co-expression network analysis (WGCNA) to identify DEGs for seed color in rapeseed

**Figure 6:**
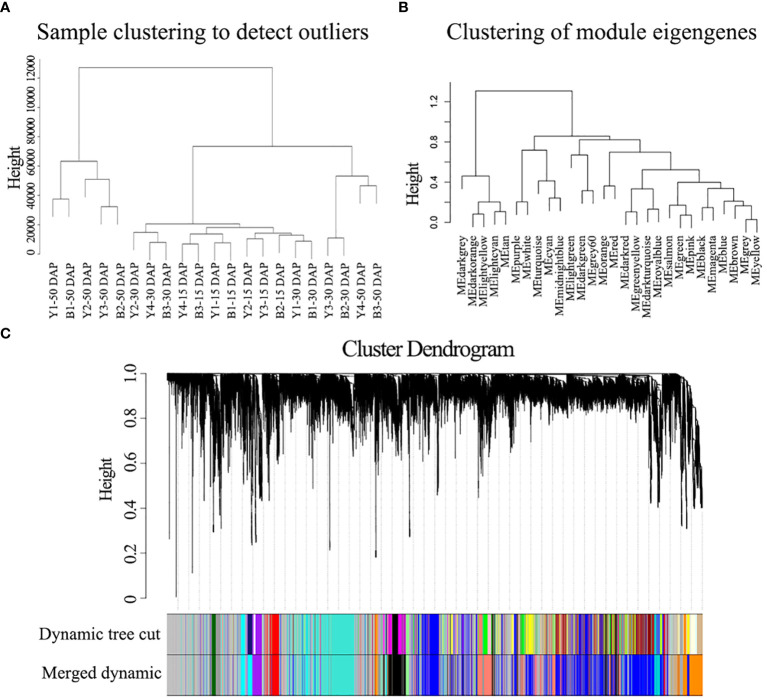
WGCNA of the gene expression matrix in seeds. **(A)** Clustering tree analysis of different samples. **(B)** Clustering tree analysis of different modules. **(C)** Clustering dendrogram of the average network adjacency for the identification of gene co-expression modules.

To further identify the genes/functional pathways associated with seed color, the weighted gene co-expression networks of yellow- and black-seeded rapeseed were constructed using RNA-seq data in this study. To reduce noise, genes with FPKM ≤ 3 in each sample were excluded. The co-expression networks were constructed based on pairwise correlations (>0.8) and minModuleSize = 250. A total of 15 distinct modules labeled with different colors were identified, with the number of genes per module ranging from 258 (orange) to 13,402 (gray) (**Figure 6**).

The hub genes detected by PPI network analysis belonged to four modules (**Table 2**). Most hub genes (23/28) were classified into the orange module, which were significantly enriched in the GO terms sulfur compound biosynthetic process, aromatic amino acid family metabolic process, and sulfur compound metabolic process. The blue and red modules each contained two hub genes, which were strongly enriched in the GO terms nuclear processes and macromolecule biosynthetic process, respectively. The salmon module, with one hub gene, was significantly enriched in protein transport, cellular macromolecule localization, and protein localization (**Table S6**). These results indicate that the modules containing hub genes are most strongly correlated with seed color, thus representing suites of interconnected genes underlying the regulation of seed color formation.

**Table 2:** WGCNA of DEGs associated with the flavonoid biosynthesis pathway in rapeseed.

Gene ID	Annotation	WGCNA
BnaA03g39500D	Bn_TT16a	blue
BnaC06g08390D	Bn_TT1a	blue
BnaC06g26980D	Bn_4CL3a	orange
BnaA07g25210D	Bn_4CL3b	orange
BnaA03g60670D	Bn_BANb	orange
BnaC01g29820D	Bn_BANc	orange
BnaC04g18950D	Bn_BANd	orange
BnaA03g14010D	Bn_C4Ha	orange
BnaA04g17570D	Bn_C4Hb	orange
BnaA05g11950D	Bn_C4Hc	orange
BnaC03g16960D	Bn_C4Hd	orange
BnaC04g14330D	Bn_C4He	orange
BnaC09g50050D	Bn_CHILa	orange
BnaA10g25120D	Bn_CHILb	orange
BnaA09g31780D	Bn_F3Ha	orange
BnaC01g14310D	Bn_LDOXa	orange
BnaC07g37670D	Bn_LDOXb	orange
BnaA03g45610D	Bn_LDOXc	orange
BnaC06g17050D	Bn_TT12a	orange
BnaA03g04590D	Bn_TT4a	orange
BnaA10g19670D	Bn_TT4b	orange
BnaC02g05070D	Bn_TT4c	orange
BnaC03g06120D	Bn_TT4d	orange
BnaC09g43250D	Bn_TT4e	orange
BnaA09g22810D	Bn_TT8a	orange
BnaA01g36200D	Bn_BANa	red
BnaC09g24870D	Bn_TT8b	red
BnaC06g32180D	Bn_MYBL2	salmon

WGCNA, the module to which the gene belongs based on WGCNA.

### The integrated gene regulatory network is linked to the flavonoid biosynthesis pathway in rapeseed

**Figure 7:**
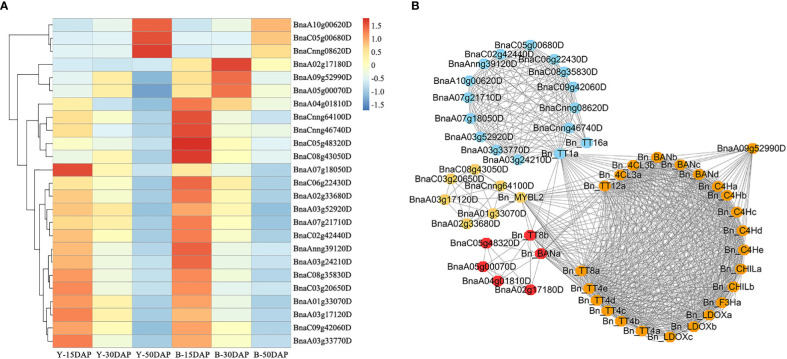
Expression profiles of 25 candidate TF genes. **(A)** Comparative expression analysis of 25 TF genes associated with seed color between yellow- and black-seeded rapeseed. The expression levels of the TF genes were calculated using FPKM values of materials with the same seed color. The heatmap was generated using the R package Pheatmap (scale = “row”, cluster_row = T). **(B)** Co-expression network of 25 TFs and hub genes. The same color indicates that genes are located in the same module, as determined by WGCNA.

Flavonoids play important roles in seed color formation and are much more complex in *Brassica* species than in the model plant Arabidopsis (Qu et al., 2016). To enhance our understanding of the regulatory gene network of the flavonoid biosynthesis pathway, an iGRN associated with *B. napus* seed color were constructed using the DEGs between the yellow- and black-seeded rapeseed. Twenty-five TFs highly interacted with hub genes involved in the flavonoid biosynthesis pathway (**Table 3**). Among genes known to be involved in the flavonoid biosynthesis pathway, the homologous genes of *KNAT7* (*BnaA09g52990D*), *NAC2* (*NAC domain containing protein 2, BnaC08g43050D* and *BnaCnng64100D*), *STK* (*BnaA03g24210D*, *BnaAnng39120D* and *BnaCnng46740D*), and *SEP2* (*BnaC05g48320D*, *BnaA01g33070D* and *BnaC09g42060D*) were expressed at higher levels in black-seeded materials, whereas TTG2 (*BnaA03g17120D* and *BnaC03g20650D*) showed the opposite pattern (**Figure 7A**). We also predicted novel TF genes, including *Integrase-type DNA-binding superfamily* (*BnaA10g00620D*, *BnaC05g00680D*, and *BnaCnng08620D*), *bZIP44* (*basic leucine-zipper 44*; *BnaA02g17180D*, *BnaC06g22430D*, and *BnaA07g21710D*), *Exonuclease family* (*BnaA05g00070D*), *SHP1* (*BnaA04g01810D*, and *BnaA07g18050D*), which showed differential expression levels between yellow- and black-seeded rapeseed (**Figure 7A**). Notably, five TF genes that were categorized in the orange and red modules by WGCNA (**Figure 7B**) had similar expression trends to most hub genes, which were closely related to the flavonoid pathway or proanthocyanidin pathway. Six TFs in the salmon module regulate the expression of the hub genes *via Bn_MYBL2* (*BnaC06g32180D*), and other TFs in the blue module play important roles in regulating the flavonoid biosynthesis pathway *via Bn_TT1a* (*BnaC06g08390D*) and *Bn_TT16a* (*BnaA03g39500D*) (**Figure 7**). These findings suggest that these TFs might be involved in regulating flavonoid pathways, thus affecting seed color in rapeseed.

**Table 3:** The predicted 25 transcription factors by iGRN.

Gene ID	AGI No.	*P*-value	Target genes	Functional description
BnaA01g33070D	AT3G02310	4.06E-05	*TT1*, *TT8*, *TT16*, *BAN*	SEPALLATA 2 (SEP2)
BnaA02g33680D	AT5G63280	8.94E-07	*TT8*, *LDOX*	C2H2-like zinc finger protein
BnaA03g17120D	AT2G37260	6.35E-06	*LDOX*	TRANSPARENT TESTA GLABRA 2 (TTG2)
BnaC03g20650D	AT2G37260	6.35E-06	*LDOX*	TTG2
BnaC08g43050D	AT3G15510	5.32E-05	*TT8*, *BAN*, *LDOX*	NAC domain containing protein 2 (NAC2)
BnaCnng64100D	AT3G15510	5.32E-05	*TT8*, *BAN*, *LDOX*	NAC2
BnaA02g17180D	AT1G75390	6.07E-05	*TT1*, *TT12*, *TT8*, *TT16*, *BAN*, *LDOX*	basic leucine-zipper 44 (bZIP44)
BnaA04g01810D	AT3G58780	1.91E-06	*TT1*, *TT12*, *TT8*, *TT16*, *BAN*	SHATTERPROOF 1 (SHP1)
BnaA05g00070D	AT2G48100	1.65E-07	*TT8*, *C4H*, *LDOX*, *PAL2*	Exonuclease family protein
BnaC05g48320D	AT3G02310	4.06E-05	*TT1*, *TT8*, *TT16*, *BAN*	SEP2
BnaA09g52990D	AT1G62990	0.00004	*TT8*, *BAN*, *LDOX*	KNOTTED-like homeobox of *Arabidopsis thaliana* 7 (KNAT7)
BnaA03g24210D	AT4G09960	0.000044	*TT1*, *TT16*, *BAN*, *F3H*	SEEDSTICK (STK)
BnaA03g33770D	AT3G15170	1.09E-05	*TT12*, *TT8*, *LDOX*	CUP-SHAPED COTYLEDON1 (CUC1)
BnaA03g52920D	AT4G34590	2.58E-06	*TT1*, *TT12*, *TT8*, *TT16*, *BAN*, *LDOX*, *F3H*	G-box binding factor 6 (GBF6)
BnaA07g18050D	AT3G58780	1.91E-06	*TT1*, *TT12*, *TT8*, *TT16*, *BAN*	SHP1
BnaA07g21710D	AT1G75390	6.07E-05	*TT1*, *TT12*, *TT8*, *TT16*, *BAN*, *LDOX*	bZIP44
BnaA10g00620D	AT1G01250	4.76E-05	*TT1*, *TT12*, *TT8*, *BAN*, *C4H*, *LDOX*	Integrase-type DNA-binding superfamily protein
BnaAnng39120D	AT4G09960	0.000044	*TT1*, *TT16*, *BAN*, *F3H*	STK
BnaC02g42440D	AT5G63280	8.94E-07	*TT8*, *LDOX*	C2H2-like zinc finger protein
BnaC05g00680D	AT1G01250	4.76E-05	*TT1*, *TT12*, *TT8*, *BAN*, *C4H*, *LDOX*	Integrase-type DNA-binding superfamily protein
BnaC06g22430D	AT1G75390	6.07E-05	*TT1*, *TT12*, *TT8*, *TT16*, *BAN*, *LDOX*	bZIP44
BnaC08g35830D	AT2G21320	4.75E-05	*TT8*, *LDOX*	B-box zinc finger family protein
BnaC09g42060D	AT3G02310	4.06E-05	*TT1*, *TT8*, *TT16*, *BAN*	SEP2
BnaCnng08620D	AT1G01250	4.76E-05	*TT1*, *TT12*, *TT8*, *BAN*, *C4H*, *LDOX*	Integrase-type DNA-binding superfamily protein
BnaCnng46740D	AT4G09960	0.000044	*TT1*, *TT16*, *BAN*, *F3H*	STK

## Discussion

In rapeseed, the yellow-seeded trait is desirable because it offers many advantages, including higher oil and protein content, lower pigment and polyphenol contents, and lower fiber content, compared to black-seeded varieties (Abraham and Bhatia, 1986; Simbaya et al., 1995; Tang et al., 2010). To date most yellow-seeded varieties were developed *via* the interspecific hybridization of *Brassica* species (Chen and Heneen., 1992; Liu et al., 2010; Qu et al., 2013), but the genetic stability and underlying mechanism remain unclear. As known, seed color is considered to be a typical quantitative trait. Despite numerous studies involving QTL analysis, GWAS, and candidate gene identification have been performed to elucidate the molecular mechanism underlying yellow seed coat formation in rapeseed (Liu et al., 2005; Badani et al., 2006; Xiao et al., 2007; Yan et al., 2009; Zhang et al., 2011; Stein et al., 2013; Zhang et al., 2013; Lian et al., 2017; Xie et al., 2020; Chao et al., 2022), few major QTL and candidate genes involved in seed color have been successfully cloned in rapeseed. Recently, a major yellow-seed QTL on chromosome A09 was reported to control seed color, oil content and fiber content, which includes important candidate genes (e.g., *BnaA09.JAZ1*, *BnaA09.GH3.3*, and *BnaA09.LOX3*) (Chao et al., 2022).

Numerous studies have revealed that the phenolic compounds cyanidin and procyanidins play dominant roles in seed color formation in rapeseed (Auger et al., 2009; Zhai et al., 2019). The flavonoid biosynthesis pathway is a major factor determining seed color in rapeseed. In the current study, GO enrichment and PPI analyses showed that down-regulated DEGs in yellow-seeded rapeseed were mainly associated with the proanthocyanidin biosynthetic pathway and the flavonoid biosynthetic pathway. The hub genes *TT16*, *BAN*, *TT4*, *LDOX*, *CHIL*, *F3H*, *TT12*, *4CL*, and *C4H* genes were located in four modules (**Table 2**) and were expressed at higher levels in black- vs. yellow-seeded rapeseed, especially during the middle and late stages of seed development (**Figure 5**). *BnTT12* was previously identified as a candidate gene involved in seed color formation (Chai et al., 2009) and was detected within QTL intervals for seed color in rapeseed (Wang et al., 2017); *TT16* is involved in proanthocyanidin accumulation and is expressed in endothelial cells (Chen et al., 2013). However, compared to black-seeded rapeseed, photosynthesis-related genes were more active in yellow-seeded rapeseed, which were clustered into the GO terms xenobiotic transmembrane transport (GO:0006855), xenobiotic transport (GO:0042908), transport (GO:0006810), and amino acid metabolism (GO:1901605) (**Table S7**). It appears that yellow-seeded rapeseed has more advantageous traits compared to black-seeded varieties, such as higher oil and protein contents; this notion requires further study.

The MYB-bHLH-WD40 (MBW) complex plays crucial roles in determining seed color by regulating the flavonoid biosynthesis pathway in various plant species (Appelhagen et al., 2010; Xu et al., 2014; Xu et al., 2015), but little is known about this process in rapeseed. In this study, *BnTT1* and *BnTT8* were identified as hub genes (**Table 2**). Indeed, the recently generated rapeseed mutant *tt8* and *tt2* (created by CRISPR/Cas9-mediated gene editing) has yellow seeds (Zhai et al., 2019; Xie et al., 2020). In addition, downregulating *BnTT1 via* RNA interference (RNAi) reduced the accumulation of flavonoids in seeds (Lian et al., 2017). Moreover, the ternary MBW complex (TT2-TT8-TTG1) was previously shown to function in the flavonoid biosynthesis pathway by regulating *DFR*, *LDOX*/*TT18*, *BAN*, and *TT12* expression, leading to changes in seed color (Lepiniec et al., 2006; Hichri et al., 2011; Xu et al., 2015). We also determined that *BAN*, *LDOX*, and *TT12* were more highly expressed in black- vs. yellow-seeded rapeseed. These genes shared similar expression patterns with *BnTT8* (**Figure 7**; **Table 2**), suggesting they might function together to regulate the flavonoid biosynthesis pathway.

With the development of high-throughput technologies such as genomic, transcriptomic, and proteomic profiling, numerous databases have been used to elucidate the complex networks involved in plant development and molecular responses to environmental cues. The release of *B. napus* datasets (Shen et al., 2015; Song et al., 2020) has made it possible to reveal the flavonoid biosynthetic pathway in rapeseed at the genome-wide level. iGRN analysis is helpful for uncovering the regulatory mechanisms of TFs in the flavonoid biosynthetic pathway in rapeseed using a network-based approach based on supervised learning for large-scale functional data integration (De Clercq et al., 2021). In the current study, 25 TF genes were identified that could be involved in regulating the genes associated with the flavonoid biosynthesis pathway based on iGRN (**Figure 7**; **Table 3**). Of these genes, *KNAT7*, *NAC2*, and *SEP2* are associated with the development of secondary cell walls in the seed coat (Grallert et al., 1997; Kunieda et al., 2008; Li et al., 2011), *KNAT7* is related to anthocyanin and proanthocyanidin biosynthesis (Bhargava et al., 2013), and *SEP2* is co-expressed with multiple MYB- and bHLH-related genes (Ahmad et al., 2022). Furthermore, *SHP1* and *CUC1* are involved in seed development (Kusumanjali et al., 2012; Mizzotti et al., 2012; Cucinotta et al., 2018). In addition, *GBF6*, *STK*, and *TTG2* are candidate genes involved in seed coat color. The expression pattern of *GBF6* is similar to that of *PAP1*, which regulates the anthocyanin pathway in Arabidopsis (Gonzalez et al., 2008; Adamiec et al., 2011). *STK* is a key gene involved in proanthocyanidin production (Mizzotti et al., 2014), and TTG2 regulates genes in the PA biosynthetic branch of the flavonoid pathway (Gonzalez et al., 2016). Therefore, we demonstrated that iGRN can provide accurate and reliable information about protein structure and function.

In addition, some novel regulators could be involved in seed coat color, including *BnbZIP44* (*BnaA02g17180D*, *BnaC06g22430D*, and *BnaA07g21710D*), Exonuclease family protein (*BnaA05g00070D*), and *SHP1* (*BnaA04g01810D*), which were more highly expressed in black-vs. yellow-seeded *B. napus* (**Figure 7**). *bZIP44* is closely related to external environmental stimuli; its knockout lines showed slower germination during seed coat rupture (Weltmeier et al., 2009; Iglesias-Fernandez et al., 2013). Recently, *bZIP44* was considered as key candidate transcription factors associated with anthocyanin biosynthesis in *C*. *maximowiczii* fruits (Zhang et al., 2023) *SHP1* is a key gene that regulates inner seed coat development in Arabidopsis (Ehlers et al., 2016). Therefore, these identified TFs can be the targets for further molecular characterization.

In conclusion, the present study is the comprehensive report on transcriptome profiling of developing seeds in yellow- and black-seeded rapeseed. A total of 1206 and 276 DEGs involved in seed coat color could be identified in the middle and late stages, and the downregulated DEGs are mainly enriched for the phenylpropanoid and flavonoid biosynthesis pathways. Moreover, twenty-five TFs involved in seed color are predicted by iGRN and WGCNA that are associated with regulating flavonoid biosynthesis pathway. Our findings provide many clues for elucidating the regulatory networks of the flavonoid biosynthesis pathway and for better understanding the molecular mechanism of the yellow-seeded trait in rapeseed.

## Data availability statement

The original contributions presented in the study are publicly available. This data can be found here: NCBI Bioproject PRJNA931458. (Pending release on acceptance).

## Author contributions

JL and CQ conceived the project and designed the experiment plans; MG, XS, SC, YW, YT, TZ, LG, and FS conducted the experiments; MG, HZ, KL, and NY analyzed the data and wrote the article; MG, HZ, NY, JL and CQ reviewed and edited the manuscript. All authors contributed to the article and approved the submitted version.
