# Supplementary material for: Comparative transcriptome analysis identifies candidate genes related to seed coat color in rapeseed
Source: Front Plant Sci. 2023 Mar 9;14:1154208. doi: 10.3389/fpls.2023.1154208 (PMC10042178; doi:10.3389/fpls.2023.1154208)
Supplement: Supplementary file 1 [file Image_1.pdf]

Supplementary Material

Comparative transcriptome analysis identifies candidate genes related to seed coat color in rapeseed

Mingwei Guan[1,2,3], Xiangtian Shi[1,2,3], Si Chen[1,2,3], Yuanyuan Wan[1,2,3], Yunshan Tang[1,2,3], Tian Zhao[1,2,3], Lei Gao[1,2,3], Fujun Sun[1,2,3], Nengwen Yin[1,2,3], Huiyan Zhao[1,2,3], Kun Lu [1,2,3], Jiana Li [1,2,3,]*, Cunmin Qu[1,2,3,]*

*** Correspondence:** Jiana Li (ljn1950@swu.edu.cn); Cunmin Qu: (drqucunmin@swu.edu.cn)

1 Supplementary Figures and Tables

1.1 Supplementary Figures

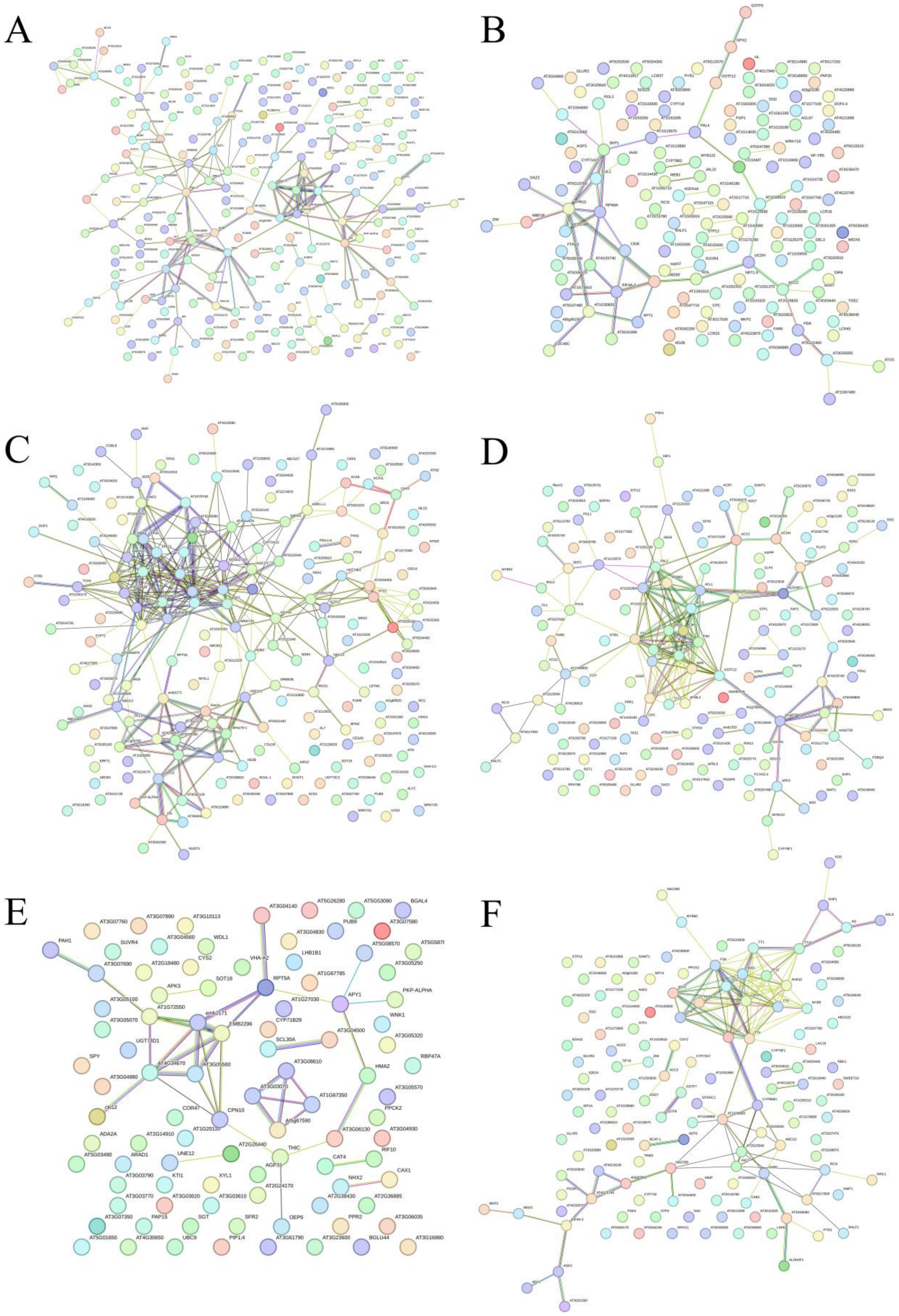
A
B
C
D
E
F
AT3G07760
AT3G07890
AT3G10113
AT3G04140
AT5G26280
AT5G53090
BGAL4
PAH1
SUVR4
AT3G04560
WDL1
AT3G04830
PUB9
AT3G07580
AT2G18480
CYS2
VHA-A2
LHB1B1
AT5G08570
AT3G07690
SOT18
RIPT5A
AT1G67785
AT3G05250
APK3
PKP-ALPHA
AT3G05100
AT1G72550
AT1G27030
APY1
emb2171
EMB2296
CYP71B29
WNK1
AT3G05070
SCL30A
AT3G04500
UGT74D1
AT3G05320
SPY
AT4G34870
AT3G05560
AT3G08610
RBP47A
AT3G04980
HMA2
AT3G03070
AT1G67350
AT3G05570
ckl12
COR47
CPN10
At5g67590
PPCK2
ADA2A
AT1G20120
AT3G06130
AT3G04930
AT2G14910
AT2G26440
THIC
RIF10
AT5G03490
ARAD1
UNE12
AGP31
CAT4
CAX1
AT3G03790
KTI1
XYL1
AT2G24170
NHX2
AT3G03770
AT3G03620
AT3G03610
AT2G38430
AT2G36885
AT3G07350
PAP15
SGT
SFR2
OEP6
PPR2
AT3G06035
AT5G01650
AT4G30650
UBC9
PIP1;4
AT3G61790
AT3G23600
BGLU44
AT3G16880

Supplementary Figure 1. PPI network of up-regulated and down-regulated DEGs in yellow seed at three different development stages. A to F, the PPI results of up-regulated DEGs in yellow seeds at 15 DAP; the PPI results of down-regulated DEGs in yellow seeds at 15 DAP; the PPI results of up-regulated DEGs in yellow seeds at 30 DAP; the PPI results of down-regulated DEGs in yellow seeds at 30 DAP; the PPI results of up-regulated DEGs in yellow seeds at 50 DAP; the PPI results of down-regulated DEGs in yellow seeds at 50 DAP.
